# Fluorescence of Half-Twisted 10-Acyl-1-methyltetrahydrobenzoquinolines

**DOI:** 10.3390/molecules29133016

**Published:** 2024-06-25

**Authors:** Christopher Abelt, Ian Day, Junkai Zhao, Robert Pike

**Affiliations:** Department of Chemistry, College of William and Mary, Williamsburg, VA 23185, USA; iday01@wm.edu (I.D.); jzhao12@wm.edu (J.Z.); rdpike@wm.edu (R.P.)

**Keywords:** solvato-chromism, charge transfer, PICT, TICT, peri

## Abstract

The steric interference of proximal dialkyl amino and acyl groups at the peri (1,8) positions of naphthalene affects the intramolecular charge transfer fluorescence. Previous studies indicate that acyl and freely rotating dimethyl amino groups twist toward coplanarity with the naphthalene ring in the excited state. The present study examines the effect of constraining the amino group in a ring. The photophysical properties of 2,2-dimethyl-1-(1-methyl-1,2,3,4-tetrahydrobenzo[*h*]quinolin-10-yl)propan-1-one (**4**), ethyl 1-methyl-1,2,3,4-tetrahydrobenzo[*h*]quinoline-10-carboxylate (**5**), and 1-methyl-1,2,3,4-tetrahydrobenzo[*h*]quinoline-10-carbaldehyde (**6**) are compared with the dimethyl amino derivatives **2** and **3.** Crystal structures of **4**–**6** show that the amine ring adopts a chair conformation, where the *N*-methyl group is axial. Computational results suggest that the pyramidal amino group planarizes and twists together with the acyl toward coplanarity in the excited state. The ring structure does not thwart the formation of a planar intramolecular charge transfer (PICT) state.

## 1. Introduction

**Figure 1:**
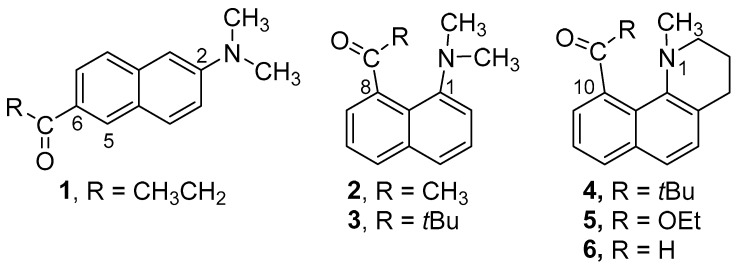
Structures of Prodan derivatives **1**–**6**.

Amino-carbonyl disubstituted naphthalenes are often highly fluorescent. They emit from an intramolecular charge transfer (ICT) excited state, where electron density has shifted from the donor amino group toward the acceptor carbonyl group. The archetypal member of this class of compounds is 6-propionyl-2-(dimethyl-amino)naphthalene (**1**, 2,6-Prodan, Figure 1) [1]. The arrangement of the donor and acceptor groups at the 2 and 6 positions gives them the maximum possible separation. The spatial separation of the charge in the excited state results in a dipole moment that is almost twice that of the ground state. Solvent molecules in the cybotactic region will reorient to stabilize the new charge distribution. Solvents with a higher polarity will stabilize the excited state better, leading to longer-wavelength emission (solvato-chromism) [2,3,4,5]. Similar solvato-chromism is observed with the 1,5- and even the 1,8-disubstituted derivatives. In the simplest examples, the substituents can rotate about the bond to the naphthalene and modulate the degree of conjugation.

Because charge transfer is integral to the behavior of these systems, the preferred geometry of the groups is important. For a related compound, dimethyl-amino-benzonitrile (DMABN), intramolecular charge transfer occurs from the amino group to the *para*-cyano group. The fluorescence behavior of model compounds suggests that the amino group twists 90° in the excited state, giving rise to a twisted intramolecular charge transfer (TICT) excited state [6,7,8,9,10]. Electronic de-coupling of the pi-systems provides maximum charge separation. Several donor–acceptor-substituted fluorophores possessing a dialkylamino group as the donor are thought to form twisted intramolecular charge transfer (TICT) excited states [11,12,13]. We have found that 2,6-, 1,5-, and 1,8-Prodan derivatives likely have planar intramolecular charge transfer (PICT) excited states [14,15,16,17]. Model compounds that prevent the rotation of the dialkyl-amino groups give the same ICT behavior as the unconstrained parent compounds.

The 1,8-derivatives of Prodan reported by Kiefl have unusual structural features [18]. If large enough, substituents at these positions will interact sterically with each other and will twist about the bond to the naphthalene to minimize the steric strain [19,20,21,22,23]. Our previous paper showed that when the carbonyl and amino groups are constrained to be nearly coplanar, the ICT emission was similar to the freely rotating derivative **2**. Given the importance of the geometry of the donor and acceptor groups, we wondered how the behavior of these systems would be affected if only the rotation of the amino portion was constrained. To this end, we prepared **4**–**6** (Figure 1) and compared their photophysical behavior to **2**–**3**.

## 2. Results

### 2.1. Structural Studies

#### 2.1.1. X-ray Structures

**Figure 2:**
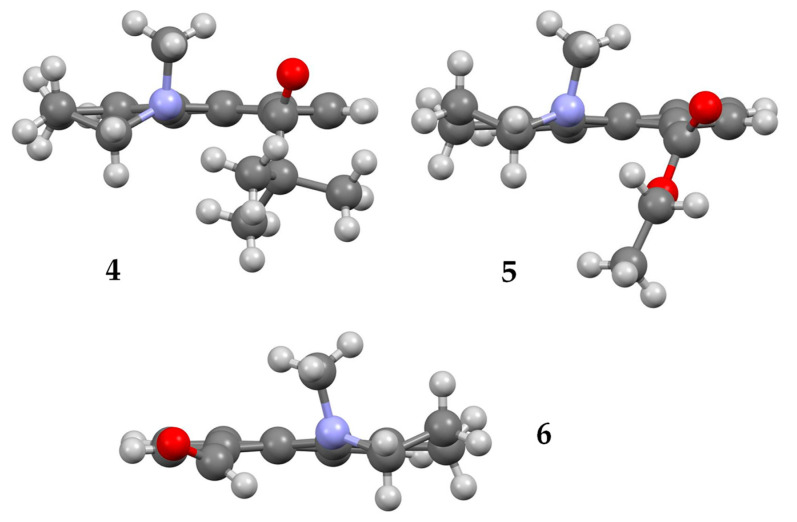
X-ray structures of **4** (**top left**), **5** (**top right**), and **6** (**bottom**). Atom, color: H, white; C, gray; N, blue; O, red.

Compounds **4**, **5**, and **6** formed crystals upon slow evaporation of a hexane/ethyl acetate solution and gave resolved crystal structures (Figure 2). The three structures display some common features. The piperidine ring has a pseudo-chair shape, and the *N*-methyl group is in an axial orientation. The carbonyl oxygen points in the same direction as the *N*-methyl group. While the carbonyl groups in **4** and **5** are twisted nearly 90° to the naphthalene ring, the carbonyl group of **6** is almost coplanar. Nevertheless, the carbonyl group and *N*-methyl group remain co-directional in **6**. Unlike the dimethyl amino derivatives **1** and **2**, the amino groups in **4**–**6** do not have two equivalent substituents. This inequivalence means that the carbonyl group can be ‘syn’ or ‘anti’ to the *N*-methyl group, or more specifically (*R*_a_,*R*_a_), (*S*_a_,*S*_a_) or (*R*_a_,*S*_a_), (*S*_a_,*R*_a_), since the conformations are axially chiral along both the C_aryl_-C=O and C_aryl_-N bonds. The crystal structures in Figure 2 are in the (*R*_a_,*R*_a_) configuration for **4** and **5**, and (*S*_a_,*S*_a_) for **6**.

#### 2.1.2. NMR Studies

**Figure 3:**
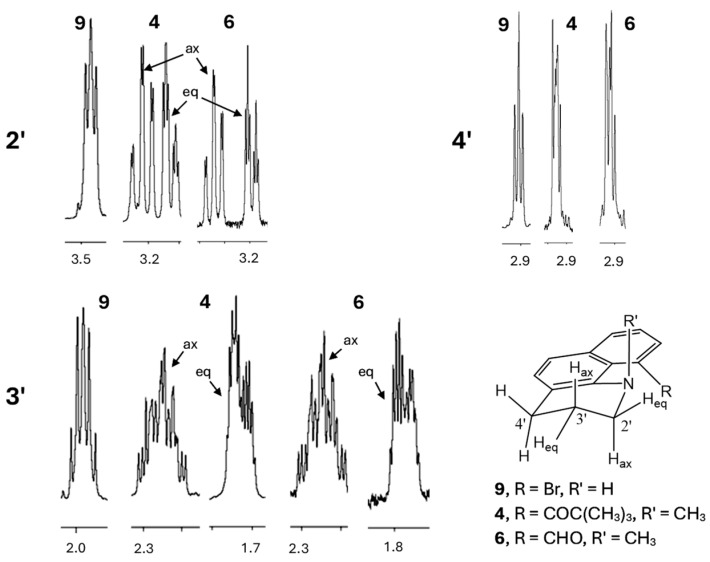
Partial ^1^H NMR spectra of **4**, **6**, and **9**, showing the saturated methylene resonances for C2′–C4′ between 1.8 and 3.5 ppm.

Proton NMR spectra corroborate the pseudo-chair conformations of the piperidine rings in **4**–**6** and **10** (Figure 3, showing only **4** and **6**). The N and the other peri position must be substituted for the chair to be rigid. In compound **9**, where H is attached to the N instead of a methyl group, conformational interconversion results in an apparent triplet (2′), quintet (3′), triplet (4′) pattern (A_2_M_2_X_2_). With *N*-methyl substituents, the 2′ and 3′ methylenes display significant diastereotopicity (e.g., **4** and **6**, Figure 3). The pseudo-axial hydrogens on C2′ and C3′ show two large coupling constants for the geminal and trans-diaxial interactions, whereas the pseudo-equatorial hydrogens show just the large geminal coupling. Proton and carbon NMR spectra of **4**–**6** are included in the Supplementary Information.

The piperidine rings in **4**–**6** are rigid. Heating a solution of **4** in CD_3_CN to 68 °C does not give any hint of broadening, much less coalescence for the diastereotopic hydrogens. Heating a solution of **10** in CDCl_3_ to 62 °C shows very slight broadening of the saturated ring hydrogens. While the piperidine ring remains fixed, the carbonyl groups still might be able to rotate about the C_aryl_-C=O bond. Kiefl characterized the rotation of the carbonyl groups in the analogous *N*,*N*-dimethyl derivatives **1** and **2** using dynamic NMR [18]. The Computational results indicate the preference for the *N*-methyl group to be aligned with the carbonyl group. Of the three carbonyl groups, the carboethoxy group in **5** is calculated to have the lowest energy for the (*R*_a_,*S*_a_), (*R*_a_,*S*_a_) rotamer (vide infra). Heating a toluene-d_8_ sample of **5** to 110 °C did not give any indication for this rotamer. The difference in energy between the rotamers is significant enough that dynamic NMR would be ineffective in revealing rotation.

### 2.2. Photophysical Studies

#### 2.2.1. Absorption

The long-wavelength absorption maxima for **4**–**6** are at 312 ± 2, 324 ± 2, and 331 ± 2 nm, respectively, over a series of solvents ranging from apolar, aprotic to polar, protic (toluene, dichloromethane, acetone, and ethanol). For comparison, **2** and **3** show absorbance maxima at 304 and 303 nm [17]. Absorption spectra are shown in Figures S1–S3. Thus, one effect of the ring structure is to shift the absorption maxima to slightly longer wavelengths. The explanation for these differences is discussed in the computational results section.

#### 2.2.2. Fluorescence

**Figure 4:**
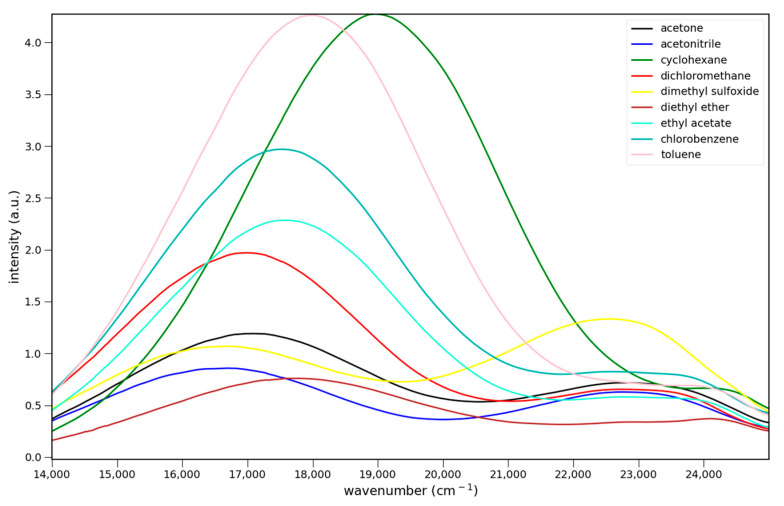

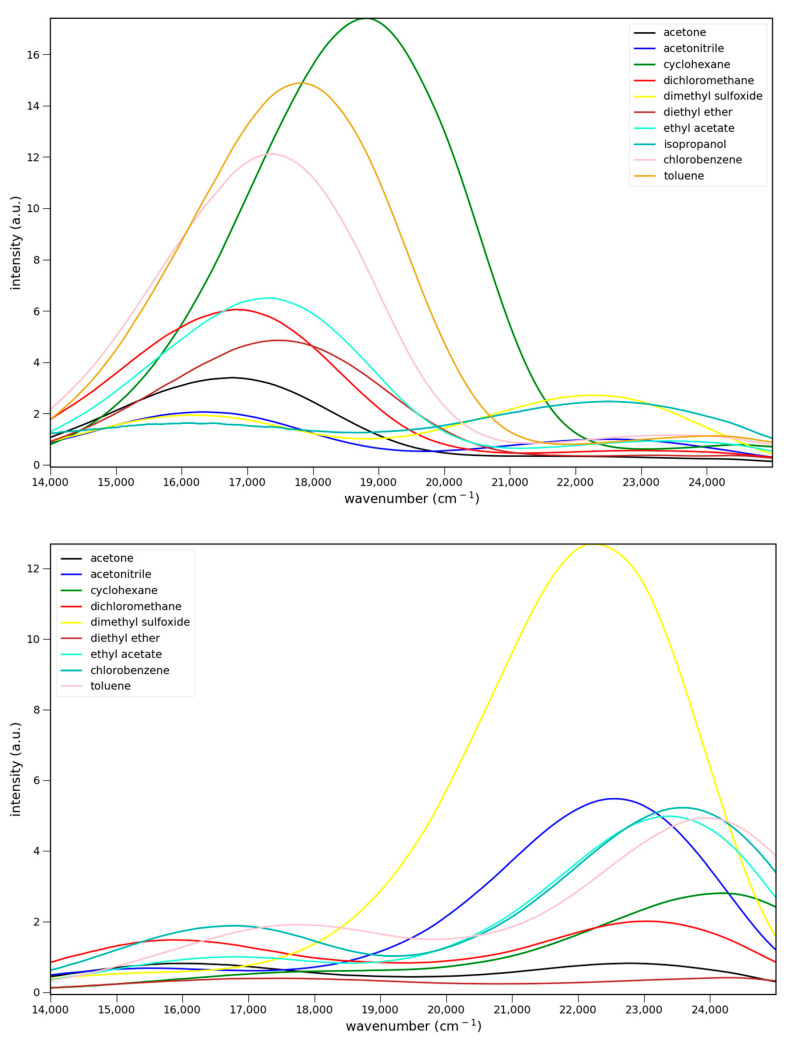
Fluorescence spectra of 7.6 × 10^−5^ M **4** (**top**), 5.5 × 10^−5^ M **5** (**middle**) and 5.1 × 10^−5^ M **6** (**bottom**) in various solvents: **▬** cyclohexane, toluene, chlorobenzene, methylene chloride, ethyl acetate, diethyl ether, acetone, acetonitrile, and dimethyl sulfoxide. Excitation at 365 nm.

**Figure 5:**
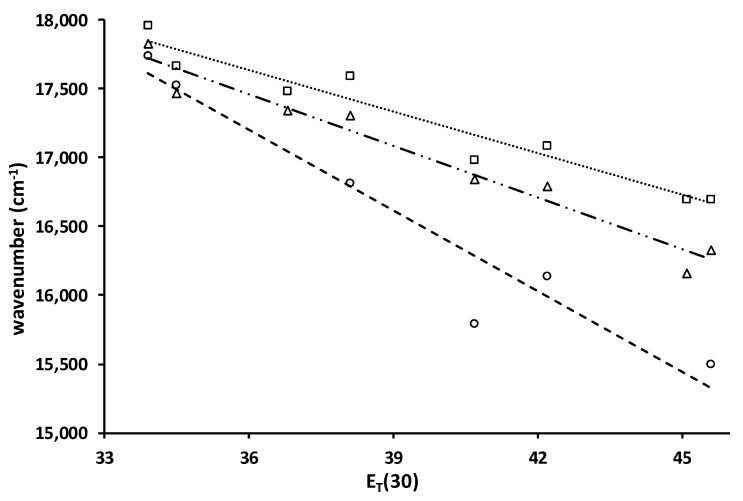
Plots of the ICT emission maxima (cm^−1^) vs. E_T_(30) for **4** (□, ····), **5** (∆, - - -) and **6** (o, — · · —) and lines of best fit, slopes, *R*^2^: −101, 0.94; −125, 0.96; −196, 0.93. Solvents are toluene, chlorobenzene, dichloromethane, ethyl acetate, diethyl ether, acetone, acetonitrile, and dimethyl sulfoxide.

The fluorescence spectra of **4**–**6** in various solvents are shown in Figure 4. All three show charge transfer emission. For Figure 4 and Figure 5 the strongest fluorescence occurs in cyclohexane, the least polar solvent in the solvent series. The emission intensities decrease with increasing solvent polarity. This decrease has been attributed to enhanced non-radiative deactivation due to the smaller energy gap between the ground and excited states in these charge transfer systems [14]. All show dual emission from a higher energy, possibly locally excited (LE), state. Emission from this state predominates in 6, and is strongest in DMSO for all three.

Relative quantum yields were determined in toluene using anthracene (Φ = 0.30) as a reference. This method gives values of 0.02 ± 0.01, 0.06 ± 0.01, and 0.02 ± 0.01, respectively, for **4**–**6**. The quantum yields in Table 1 are based on the toluene value after adjusting for the differences in refractive indices (*n*^2^_solvent_/*n*^2^_toluene_) [24]. These values are similar to those for **2** and **3**, 0.08 and 0.03, respectively, in toluene.

#### 2.2.3. Solvato-Chromism

Solvato-chromism in acyl naphthylamines results from charge transfer from the nitrogen donor to the carbonyl acceptor. Because more polar solvents can better stabilize a greater charge, the emission shifts to lower energy with increasing solvent polarity. The plot of the fluorescence maxima vs. the E_T_(30) solvent polarity parameter serves as a measure of the magnitude of the solvato-chromism (Figure 4) [25]. A steep negative slope indicates strong solvent stabilization of the excited state. The slopes of the plots for **4** and **5** are similar in magnitude. The standard deviation of the slopes is 11%. By comparison, the slopes for **2** and **3** are −216 and −177, respectively, which are in line with **6**. The ring structure reduces the size of the solvato-chromism. The Stokes shifts for **4**–**6** are 15,400, 14,500, and 14,700 cm^−1^, respectively, in acetonitrile. The Stokes shifts for **2**–**3** in acetonitrile are 16,300 and 15,400 cm^−1^. The two pivaloyl derivatives, **3** and **4**, give identical Stokes shifts. While the absorption of **4** is at a longer wavelength than **3**, its emission is also at a longer wavelength (599 vs. 567 nm).

### 2.3. Computational Studies

**Figure 6:**
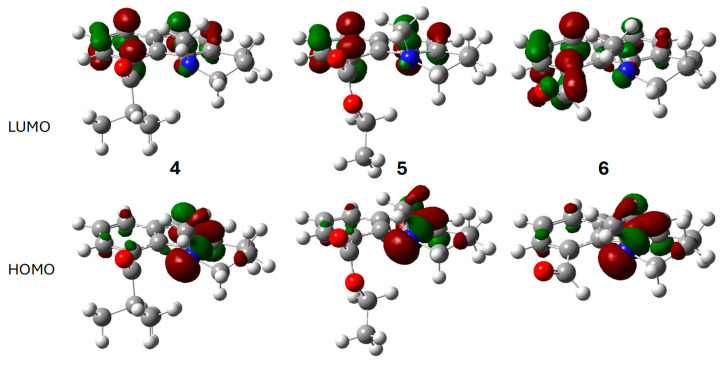
Calculated frontier molecular orbitals for the ground states of **4** (**left**), **5** (**middle**), and **6** (**right**): LUMO (**top row**), HOMO (**bottom row**). Atom, color: H, white; C, gray; N, blue; O, red. Molecular orbital phases are dark red and green.

The optimized electronic structures for the ground and excited states of **4**–**6** were calculated using Gaussian 16 [26]. The frontier molecular orbitals of **4**–**6** for the gas phase ground state are shown in Figure 6. As with the dimethylamino derivatives **2** and **3**, the HOMO is principally the nitrogen lone pair for all three. The carbonyl group is a component of the LUMO only in **6**. This conjugation results in the longer wavelength absorption maximum for **6**. In **4** and **5**, the LUMO is largely the naphthalene B_2g_ LUMO. The lowest energy singlet excited state (S_1_) results mostly from a simple HOMO→LUMO transition (Table S1).

The restricted acyl group rotation and slow piperidine chair interconversion in **4**–**6** result in chiral axes along the C_aryl_-C=O and C_aryl_-N bonds and four stereoisomers: (*R*_a_,*R*_a_), (*S*_a_,*S*_a_) or (*R*_a_,*S*_a_), (*S*_a_,*R*_a_). In the first pair of enantiomers, the carbonyl group points in the same direction as the *N*-methyl group. The stereoisomers shown in Figure 6 are all (*S*_a_,*S*_a_). Rotation about the C_aryl_-C=O gives the (*R*_a_,*S*_a_), (*R*_a_,*S*_a_) pair of enantiomers. Optimization of these structures results in local energy minima that are higher energy than the (*R*_a_,*R*_a_), (*S*_a_,*S*_a_) structures by 5.1, 3.8, and 4.2 kcal/mol, respectively. These energy differences explain why dynamic NMR gives no indication of rotation about the C_aryl_-C=O bond, even with **6**, where steric hindrance is negligible. At 120 °C, ester **5**, having the smallest energy difference between the conformations, would have an equilibrium population of only 1% for the (*R*_a_,*S*_a_), (*S*_a_,*R*_a_) enantiomers (vide supra).

The calculated structures for the ground and excited states of **4**–**6** share several structural features. Most relevant are the dihedral angles about the C_aryl_-C=O (d1, d2), C_aryl_-N (d3, d4), and the central naphthalene C=C (d5, d6), shown in Table 2. If the paired dihedral angles are nearly the same, this indicates that the groups on either end of the common atoms of the dihedral angles are nearly planar. In this case, a twist angle can be calculated as the absolute value of the average of the paired dihedral angles (Table 2). The planar approximation does not hold for the C_aryl_-N bond in the ground-state structures. Here, the N atom is significantly pyramidal, and, as shown in the crystal structures, the methyl group is in an axial orientation. The difference between the paired dihedral angles is a measure of the degree of pyramidalization.

In the ground state, the carbonyl groups of **4** and **5** are twisted significantly (~75°), while in **6**, the carbonyl is twisted only half as much. The central naphthalene C=C has almost no twist (~6°). In the excited states, the planar approximation for the end groups holds for all three dihedral angle pairs in all three structures. The carbonyl groups of **5** and **6** show little twisting (~5°) and therefore are nearly coplanar with the naphthalene ring. The carbonyl group of **4** still twists significantly in the excited state (40°), but this value is nearly half that of the ground state (76°). This result is due to the steric effect of the *tert*-butyl group. In the excited states, not only have the amino groups become planar, but they show a relatively small degree of twisting from the naphthalene (~22°). Finally, the naphthalene nucleus doubles its twist (13°) about the central bond in the excited state. The torque responsible for this deformation comes from the planarization of the amino group and the resulting steric hindrance with the carbonyl group.

The calculated absorption and emission values are shown in Table 3. Both the calculated absorption and emission values are predicted to be at shorter wavelengths than the experimental values. As with **2** and **3**, the dipole moments of the ground and relaxed excited states are nearly the same, except for **5**. The solvato-chromism shown in Figure 4 indicates that the solvent stabilizes the excited states. The 2,6 and 1,5-prodan regio-isomers show similar solvato-chromism, but much larger changes in the dipole moments [14,15]. In the 1,8-donor–acceptor fluorophores, **2**–**3** and **4**–**6**, solvato-chromism likely results from an increase in charge more so than the separation of charge.

The calculated Stokes shifts in Table 3 compare favorably with the experimental ones in toluene (13,800, 13,100, and 12,300 cm^−1^, respectively). The Stokes shifts are large because the structures of the excited and ground states differ greatly.

## 3. Materials and Methods

Reagents were obtained from Acros Organics or Sigma-Aldrich unless otherwise indicated. *N*-bromosuccinimide was recrystallized from water. Proton and carbon nuclear magnetic resonance spectra were obtained with an Agilent DD2-400 spectrometer. X-ray measurements were made using graphite-monochromated Mo *K*α radiation on a Bruker-AXS three-circle Apex DUO diffractometer equipped with a SMART Apex II CCD detector. All solvents used for absorption and fluorescence were spectrophotometric grade. Absorption and fluorescence data were collected using a fiber optic system with an Ocean Optics Maya CCD detector using a miniature deuterium/tungsten lamp and a 365 nm LED light source, respectively. Cuvettes were thermostated at 23 °C for fluorescence studies. Emission intensities were processed by subtracting the electronic noise, converting wavelengths to wavenumbers, multiplying by λ^2^/λ_max_^2^ to account for the effect of the abscissa-scale transformation [24], and dividing by the spectral response of the Hamamatsu S10420 CCD. Relative quantum yields were determined using anthracene as the reference (Φ = 0.30).

Electronic structure calculations were carried out using Gaussian 16 [26]. Ground-state geometries were optimized using the DFT CAM-B3YLP method with the 6-311G + (2d,p) basis set and the IEFPCM solvent model for toluene. Excited states were optimized using the TD-SCF DFT CAM-B3LYP method with the 6-311G + (2d,p) basis set and the IEFPCM solvent model for toluene. The optimization for **5** did not converge, and the lowest energy iteration is reported.

**Scheme 1:**
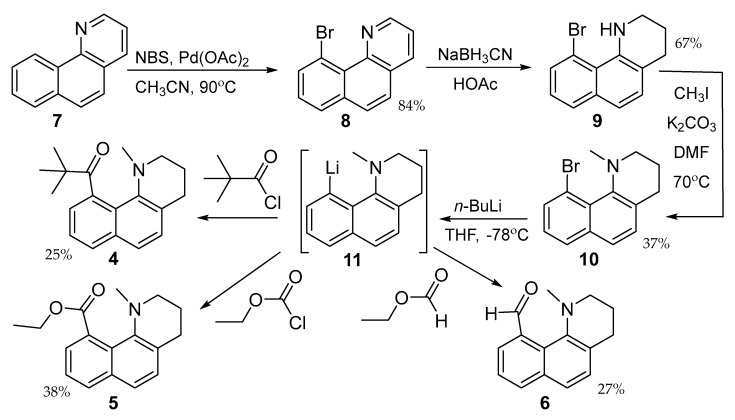
Preparation of **4**−**6** starting from **7** and proceeding through **11**.

The syntheses of **4**–**6** are shown in Scheme 1. Bromination of **7** is directed to the peri-position by the palladium catalyst [25]. Exhaustive reduction of the heteroaromatic ring with sodium cyanoborohydride followed by forcing methylation gives the common intermediate **10**. Metal halogen exchange forms the lithiated aryl species **11** and gives **4**–**6** after reaction with pivaloyl chloride, ethyl chloroformate, and ethyl formate, respectively. Reaction of **10** with carbonyl electrophiles with α-hydrogens gives only hydrogen atom abstraction.

Crystals for X-ray diffraction were grown by dissolving the compounds in a minimum amount of ethyl acetate (~1 mL), diluting with hexanes (~10 mL), and allowing the solvents to evaporate slowly.

### 3.1. 10-Bromobenzo[h]quinoline (**8**)

Benzo[*h*]quinoline (3.82 g, 21.4 mmol), *N*-bromosuccinimide (4.00 g, 22.4 mmol, recrystallized from water), and Pd(OAc)_2_ (140 mg, 0.62 mmol) were added to a 60 mL Ace-Thred pressure tube and covered with acetonitrile (30 mL). The tube was heated in an oil bath at 90 °C for 2 days with stirring. The reaction was allowed to cool to room temperature and the solids were filtered with suction and washed with CH_2_Cl_2_. The filtrate was diluted with hexanes (100 mL) and CH_2_Cl_2_ (50 mL). The organic phase was washed with 1M NaOH (2 × 100 mL), dried over Na_2_SO_4_, filtered, and concentrated in vacuo. The resulting solid (4.62 g, 17.9 mmol, 84%) is used without further purification [28].

### 3.2. 10-Bromo-1,2,3,4-tetrahydrobenzo[h]quinoline (**9**)

10-Bromobenzo[*h*]quinoline (**8**, 5.4 g, 21 mmol) in acetic acid (45 mL) was cooled in a water bath. NaBH_3_CN (2.4 g, 38 mmol) was added in portions. The cooling bath was removed and stirred at room temperature under a CaCl_2_ drying tube for several hours. More NaBH_3_CN (2.4 g, 38 mmol) was added, and stirring continued overnight. The slurry was added dropwise to 2 M NaOH (250 mL). NaCl (30 g) was added with stirring, and the liquid was decanted from the precipitated solids. The solids were washed with CH_2_Cl_2_ (75 mL) and combined with the decanted liquids. The layers were shaken together, and the CH_2_Cl_2_ layer was removed. The aq. phase was extracted once more with CH_2_Cl_2_ (75 mL). The combined CH_2_Cl_2_ layers were washed with water (2 × 100 mL), dried over CaCl_2_, and concentrated in vacuo. The resulting oil was distilled under vacuum (0.1 torr) giving **9** as a yellow oil (2.49 g, 14.1 mmol, 67%). NMR ^1^H (400 MHz, CDCl_3_): 7.62 (d, *J* = 8.1 Hz, 1H), 7.57 (d, *J* = 7.4 Hz, 1H), 7.11–7.03 (m, 3H), 6.56 (br. s. 1H), 3.47 (t, *J* = 5.5 Hz, 2H), 2.91 (t, *J* = 6.5 Hz, 2H), 1.98 (dd, *J* = 6.5, 5.5 Hz, 2H); ^13^C (400 MHz, CDCl_3_): *δ* 140.75, 136.47, 131.94, 129.69, 129.39, 125.04, 120.38, 116.70, 116.56, 116.47, 42.05, 28.68, 21.40.

### 3.3. 10-Bromo-1-methyl-1,2,3,4-tetrahydrobenzo[h]quinoline (**10**)

10-Bromo-1,2,3,4-tetrahydrobenzo[*h*]quinoline (**9**, 1.29 g, 7.96 mmol) was dissolved in DMF (15 mL). NaH (250 mg, 60% in mineral oil, 6.25 mmol) was added in small portions with stirring. After 30 min, methyl iodide (1.63 g, 11.5 mmol) was added, the flask was loosely capped, and the mixture was stirred overnight. Another portion of NaH (250 mg, 60% in mineral oil, 6.25 mmol) was added and stirred for 30 min. Methyl iodide (1.66 g, 11.7 mmol) was added and the mixture was stirred for another day. The mixture was filtered with suction. DMF was removed via vacuum distillation. Vacuum distillation of the pot residue gave **10** as a yellow oil (810 mg, 2.9 mmol, 37%, containing 8% unreacted **9**). NMR ^1^H (400 MHz, CDCl_3_): 7.68 (d, *J* = 7.4 Hz, 1H), 7.65 (d, *J* = 8.1 Hz, 1H), 7.31 (d, *J* = 8.3 Hz, 1H), 7.14–7.09 (m, 2H), 3.38–3.30 (m, 1H), 3.19–3.12 (m, 1H), 2.94–2.87 (m, 2H), 2.71, (s, 3H), 2.14–2.02 (m, 1H), 1.78–1.69 (m, 1H); ^13^C (400 MHz, CDCl_3_): *δ* 144.95, 136.20, 132.90, 128.72, 128.63, 127.02, 126.15, 125.19, 121.55, 118.07, 51.04, 46.53, 28.88, 17.11.

### 3.4. 2,2-Dimethyl-1-(1-methyl-1,2,3,4-tetrahydrobenzo[h]quinolin-10-yl)propan-1-one (**4**)

10-Bromo-1-methyl-1,2,3,4-tetrahydrobenzo[*h*]quinoline (**10**, 380 mg, 1.4 mmol) was dissolved in THF (10 mL) and cooled to −78 °C under N_2_. A 1.6 M solution of *n*-BuLi in hexanes (900 µL, 1.4 mmol) was added slowly dropwise. The reaction was stirred at −78 °C for 30 min. Freshly distilled pivaloyl chloride (200 µL, 1.6 mmol) was added dropwise, and the reaction was allowed to warm to room temperature with continued stirring. The reaction was quenched with aq. NH_4_Cl (5 mL) and diluted with hexanes (75 mL) and EtOAc (25 mL). The organic phase was washed with water (2 × 100 mL), dried over Na_2_SO_4_, and concentrated in vacuo, giving a yellow oil (320 mg). methyl-1,2,3,4-tetrahydrobenzo[*h*]quinoline was largely removed by diluting with hexanes and CH_2_Cl_2_ (1 mL ea), washing with 1 M HCl (3 × 2 mL) and water (3 × 2 mL), drying over CaCl_2_, and concentrating in vacuo. Column chromatography of the residue on silica gel with isocratic elution (5% ethyl acetate in hexanes) gave **4** (100 mg, 0.36 mmol, 25%) as a light brown oil. Crystals were obtained as above for X-ray diffraction, m.p. 41–43 °C. NMR ^1^H (400 MHz, CDCl_3_): 7.73 (d, *J* = 8.0 Hz, 1H), 7.44 (d, *J* = 8.3 Hz, 1H), 7.37 (dd, *J* = 8.0, 7.1 Hz, 1H), 7.14 (d, *J* = 8.3 Hz, 1H), 7.07 (d, *J* = 7.1 Hz, 1H), 3.27–3.09 (m, 2H), 2.95–2.89 (m, 2H), 2.42, (s, 3H), 2.32–2.17 (m, 1H), 1.79–1.68 (m, 1H), 1.03 (s, 9H); ^13^C (400 MHz, CDCl_3_): *δ* 213.64, 144.25, 137.40, 133.12, 128.68, 128.64, 128.63, 127.06, 124.79, 124.40, 123.45, 51.63, 45.78, 45.49, 27.24, 27.08, 15.71.

### 3.5. Ethyl 1-Methyl-1,2,3,4-tetrahydrobenzo[h]quinoline-10-carboxylate (**5**)

10-Bromo-1-methyl-1,2,3,4-tetrahydrobenzo[*h*]quinoline (**10**, 600 mg, 3.4 mmol) was dissolved in THF (3 mL) and cooled to −78 °C under N_2_. A 1.6 M solution of *n*-BuLi in hexanes (2.0 mL, 3.2 mmol) was added slowly dropwise. The reaction was stirred at −78 °C for 20 min. Freshly distilled ethyl chloroformate (400 mg, 3.7 mmol) was added in one portion, and the reaction was allowed to warm to 0 °C with continued stirring. The reaction was quenched with aq. NH_4_Cl (5 mL) and diluted with Et_2_O (125 mL). The organic phase was washed with water (2 × 100 mL), dried over Na_2_SO_4_, and concentrated in vacuo, giving an oil. Column chromatography of the residue on silica gel with gradient elution (0→20% ethyl acetate in hexanes) gave **5** (350 mg, 1.3 mmol, 38%) as a yellow solid, m.p. 78–80 °C. NMR ^1^H (400 MHz, CDCl_3_): 7.78 (d, *J* = 7.8 Hz, 1H), 7.45 (d, *J* = 8.3 Hz, 1H), 7.42 (d, *J* = 7.0, 1H), 7.37 (dd, *J* = 7.8, 7.0 Hz, 1H), 7.17 (d, *J* = 8.3 Hz, 1H), 4.38–4.25 (m, 2H), 3.26–3.08 (m, 2H), 2.98–2.90 (m, 2H), 2.56, (s, 3H), 2.30–2.17 (m, 1H), 1.75–1.62 (m, 1H), 1.33 (t, *J* = 7.1 Hz, 3H); ^13^C (400 MHz, CDCl_3_): *δ* 172.06, 144.48, 133.61, 130.79, 129.54, 128.68, 127.95, 126.85, 125.37, 124.32, 122.84, 61.00, 50.37, 45.43, 27.00, 15.52, 14.38.

### 3.6. 1-Methyl-1,2,3,4-tetrahydrobenzo[h]quinoline-10-carbaldehyde (**6**)

10-Bromo-1-methyl-1,2,3,4-tetrahydrobenzo[*h*]quinoline (**10**, 600 mg, 3.4 mmol) was dissolved in THF (3 mL) and cooled to −78 °C under N_2_. A 1.6 M solution of *n*-BuLi in hexanes (2.0 mL, 3.2 mmol) was added slowly dropwise. The reaction was stirred at −78 °C for 30 min. Ethyl formate (400 mg, 5.4 mmol) was added in one portion, and the reaction was allowed to warm to 0 °C with continued stirring. The reaction was quenched with aq. NH_4_Cl (5 mL) and diluted with hexanes (75 mL) and EtOAc (25 mL). The organic phase was washed with water (2 × 100 mL), dried over Na_2_SO_4_, and concentrated in vacuo, giving an oil. Column chromatography of the residue on silica gel with gradient elution (0→10% ethyl acetate in hexanes) gave **6** (220 mg, 0.92 mmol, 27%). It was sublimed under vacuum (0.1 torr) before use, giving a beige solid, m.p. 84–86 °C. NMR ^1^H (400 MHz, CDCl_3_): 10.91 (s, 1H), 7.85 (d, *J* = 8.2 Hz, 1H), 7.59 (d, *J* = 7.1 Hz, 1H), 7.46 (d, *J* = 8.5, 1H), 7.42 (dd, *J* = 8.0, 7.5 Hz, 1H), 7.20 (d, *J* = 8.5 Hz, 1H), 3.29–3.30 (m, 1H), 3.33–3.16 (m, 1H), 2.98–2.93 (m, 2H), 2.59, (s, 3H), 2.21–2.08 (m, 1H), 1.81–1.74 (m, 1H); ^13^C (400 MHz, CDCl_3_): *δ* 193.86, 144.64, 136.20, 133.80, 131.74, 128.80, 128.36, 128.04, 125.19, 124.85, 122.74, 50.73, 44.08, 27.23, 15.55.

## 4. Discussion

The groups on the peri positions of naphthalene affect the behavior of donor–acceptor-substituted fluorophores due to steric interference. For the three carbonyl groups studied here, the bulkier pivaloyl (**4**) and carboethoxy (**5**) groups twist nearly 90° from the naphthalene ring in both the crystal and calculated structures. The small formyl group (**6**) still twists slightly but is sufficiently planar for the carbonyl group to be incorporated in the LUMO, resulting in a longer-wavelength absorption. In all cases, the piperidine ring adopts a pseudo-chair conformation, where the *N*-methyl group is in an axial orientation. While an equatorial orientation is typically lower energy, an equatorial methyl group would have a strong steric interaction with the adjacent carbonyl group, even the formyl group. The axial methyl group and chair structure result in a pyramidal nitrogen atom that is conjugated poorly with the naphthalene ring. Surprisingly, the geometries of the amino groups in **4**–**6** are very similar to the freely rotating dimethyl amino groups in **2** and **3** in the ground-state structures. In the excited state, the amino group flattens and twists along with the carbonyl group to near coplanarity with the naphthalene ring. For the piperidine ring, this means that it is no longer chair-like, and the *N*-methyl is neither axial nor equatorial nor pyramidal. Given the extra constraint of the piperidine ring, it is even more surprising that both the carbonyl groups and the amino groups have nearly the same geometries in **4**–**6** as in **2**–**3**. The smaller solvato-chromism observed for **4**–**6** is likely a consequence of the extra alkyl group substitution and ring structure that affects the charge distribution and interaction with solvent molecules. Finally, the constraint imposed by the piperidine ring excludes the possibility of forming a TICT state. Chen and Fang have shown that such TICT states may be a source of non-radiative deactivation in these systems [12,13,29]. The present study offers corroboration that these systems emit from a mostly PICT state.

## Figures and Tables

**Table 1:** Fluorescence properties of **4**–**6**.

	4		5		6	
	Φ_f_	λ_em_	Φ_f_	λ_em_	Φ_f_	λ_em_^c^
Solvent ^a^	×10^−2^	(nm)	×10^−2^	(nm)	×10^−2^	(nm)
Cyc	2.6	528	7.6	531	0.5	sh ^b^
Tol	2.4	557	6.0	561	1.9	564
PhCl	1.5	572	4.5	576	0.1	sh
CH_2_Cl_2_	0.9	589	2.2	594	1.0	633
EtOAc	1.2	568	2.4	578	0.9	595
Et_2_O	0.4	566	1.8	572	0.3	571
Me_2_CO	0.5	585	1.2	596	0.1	620
MeCN	0.3	599	0.7	612	2.4	645
DMSO	0.4	599	0.5	619	1.0	sh

^a^ Solvents are cyclohexane, toluene, chlorobenzene, dichloromethane, ethyl acetate, diethyl ether, acetone, acetonitrile, and dimethyl sulfoxide. ^b^ Shoulder. ^c^ ICT band.

**Table 2:** Dihedral and twist angles for the calculated ground and excited states of **4**–**6**.

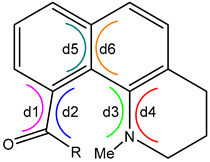			d1	d2	twist	d3	d4	twist	d5	d6	twist
	**4**	S_1_	−35	−46	40	−27	−21	24	13	18	16
		S_0_	−72	−81	76	−76	−25	50	6	6	6
	**5**	S_1_	−7	2	2	−19	−25	22	11	11	11
		S_0_	−71	−77	74	−77	−25	51	6	6	6
	**6**	S_1_	−9	−5	7	−29	−23	26	12	13	12
		S_0_	−45	−43	44	−74	−25	49	6	8	7

**Table 3:** Computed emission (em) and absorption (abs) maxima, dipole moments (em: S_1_, abs: S_0_), and Stokes shifts for **4**–**6** (top) and the related experimental values in toluene (bottom) *^a^*.

		4		5		6	
		em	abs	em	abs	em	abs
calculated	λ_max_ (nm)	488	294	518	300	511	317
	*k*_r_ × 10^7^ (s^−1^) *^b^*	2.7		2.3		2.2	
	μ (D)	3.6	3.5	3.8	5.4	4.9	4.7
	Stokes shift (cm^−1^)	13,500		14,100		12,000	
experimental							
	λ_max_ (nm)	557	315	561	323	564	333
	Φ (em), log ε (abs)	0.024	4.0	0.060	3.9	0.019	3.9
	Stokes shift (cm^−1^)	13,800		13,100		12,300	

*^a^* Φ is the relative quantum yield and ε is the molar absorptivity (M^−1^ cm^−1^). *^b^* Radiative rate calculated as *f*_osc_ ×ΔE^2^/1.5, where *f*_osc_ is the oscillator strength for the emission [27].

## Data Availability

The data presented in this study are available either in this article or in the Supplementary Materials.

## Supplementary Materials

The following supporting information can be downloaded at https://www.mdpi.com/article/10.3390/molecules29133016/s1: crystallographic information files (CIF) for compounds **4**–**6**; checkCIF report from the Cambridge Crystallographic Data Centre (CCDC); crystal structure and refinement data for **4**–**6**; bond distances and angles for **4**–**6**; absorption spectra for **4**–**6**; proton and carbon NMR spectra for **4**–**6**; and Gaussian input files (GJF) with their energies for the relaxed first singlet excited states of **4**–**6**.
