# Supplementary material for: Fluorescence of Half-Twisted 10-Acyl-1-methyltetrahydrobenzoquinolines
Source: Molecules. 2024 Jun 25;29(13):3016. doi: 10.3390/molecules29133016 (PMC11243386; doi:10.3390/molecules29133016)
Supplement: Supplementary file 1 [file molecules-29-03016-s001.zip › molecules-3040043-supplementary.pdf]

Fluorescence of Half Twisted 10-Acyl-1-methyltetrahydrobenzoquinolines

Christopher Abelt*, Ian Day, Junkai Zhao, and Robert Pike

College of William and Mary, Department of Chemistry, Williamsburg, Virginia, 23185, USA

Supplementary Information

Table of Contents

Title and Table of Contents S1
X-ray crystal structure data S2
Bond lengths [Å] and angles [°] for Compound **4** S2
Bond lengths [Å] and angles [°] for Compound **5** S4
Bond lengths [Å] and angles [°] for Compound **6** S6
Crystal data and structure refinement for Compound **4** S7
Crystal data and structure refinement for Compound **5** S8
Crystal data and structure refinement for Compound **6** S9
Absorption spectroscopy S5
Table S1. Calculated excitation data for **4-6** S10
Figure S1. Absorption spectra of **4** S10
Figure S2. Absorption spectra of **5** S11
Figure S3. Absorption spectra of **6** S11
NMR spectroscopy S12
Figure S4. ^{1}H and ^{13}CNMR spectra of **4**. S12
Figure S5. ^{1}H and ^{13}CNMR spectra of **5** S13
Figure S6. ^{1}H and ^{13}CNMR spectra of **6** S14

Bond lengths [Å] and angles [°] for Compound **4**.

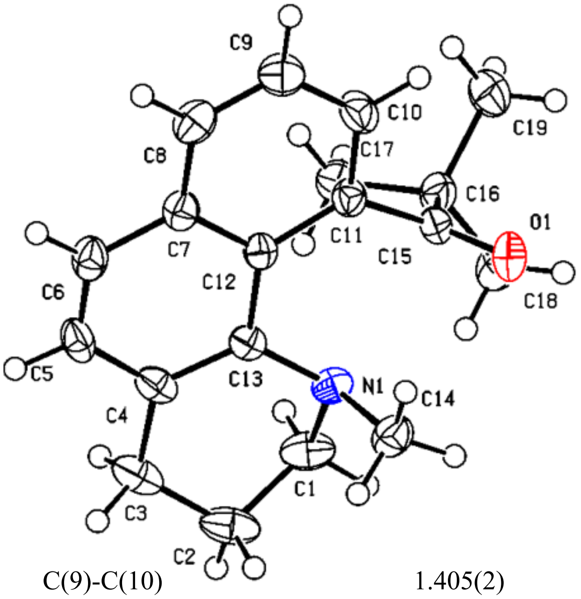

O(1)-C(15)	1.218(2)	C(9)-C(10)	1.405(2)
N(1)-C(13)	1.425(2)	C(9)-H(9)	0.9500
N(1)-C(14)	1.471(2)	C(10)-C(11)	1.377(2)
N(1)-C(1)	1.474(2)	C(10)-H(10)	0.9500
C(1)-C(2)	1.525(3)	C(11)-C(12)	1.422(2)
C(1)-H(1A)	0.9900	C(11)-C(15)	1.517(2)
C(1)-H(1B)	0.9900	C(12)-C(13)	1.429(2)
C(2)-C(3)	1.509(3)	C(14)-H(14A)	0.9800
C(2)-H(2A)	0.9900	C(14)-H(14B)	0.9800
C(2)-H(2B)	0.9900	C(14)-H(14C)	0.9800
C(3)-C(4)	1.509(2)	C(15)-C(16)	1.542(2)
C(3)-H(3A)	0.9900	C(16)-C(18)	1.524(2)
C(3)-H(3B)	0.9900	C(16)-C(17)	1.525(2)
C(4)-C(13)	1.384(2)	C(16)-C(19)	1.544(2)
C(4)-C(5)	1.416(2)	C(17)-H(17A)	0.9800
C(5)-C(6)	1.354(2)	C(17)-H(17B)	0.9800
C(5)-H(5)	0.9500	C(17)-H(17C)	0.9800
C(6)-C(7)	1.412(2)	C(18)-H(18A)	0.9800
C(6)-H(6)	0.9500	C(18)-H(18B)	0.9800
C(7)-C(8)	1.420(2)	C(18)-H(18C)	0.9800
C(7)-C(12)	1.424(2)	C(19)-H(19A)	0.9800
C(8)-C(9)	1.359(3)	C(19)-H(19B)	0.9800
C(8)-H(8)	0.9500	C(19)-H(19C)	0.9800

C(13)-N(1)-C(14)	111.98(12)	C(14)-N(1)-C(1)	111.19(13)
C(13)-N(1)-C(1)	111.99(13)	N(1)-C(1)-C(2)	112.78(14)

N(1)-C(1)-H(1A)	109.0	C(11)-C(10)-H(10)	119.3
C(2)-C(1)-H(1A)	109.0	C(9)-C(10)-H(10)	119.3
N(1)-C(1)-H(1B)	109.0	C(10)-C(11)-C(12)	119.80(14)
C(2)-C(1)-H(1B)	109.0	C(10)-C(11)-C(15)	115.56(14)
H(1A)-C(1)-H(1B)	107.8	C(12)-C(11)-C(15)	124.59(13)
C(3)-C(2)-C(1)	110.14(14)	C(11)-C(12)-C(7)	118.57(13)
C(3)-C(2)-H(2A)	109.6	C(11)-C(12)-C(13)	122.52(13)
C(1)-C(2)-H(2A)	109.6	C(7)-C(12)-C(13)	118.88(14)
C(3)-C(2)-H(2B)	109.6	C(4)-C(13)-N(1)	122.84(14)
C(1)-C(2)-H(2B)	109.6	C(4)-C(13)-C(12)	120.36(14)
H(2A)-C(2)-H(2B)	108.1	N(1)-C(13)-C(12)	116.79(13)
C(4)-C(3)-C(2)	113.13(15)	N(1)-C(14)-H(14A)	109.5
C(4)-C(3)-H(3A)	109.0	N(1)-C(14)-H(14B)	109.5
C(2)-C(3)-H(3A)	109.0	H(14A)-C(14)-H(14B)	109.5
C(4)-C(3)-H(3B)	109.0	N(1)-C(14)-H(14C)	109.5
C(2)-C(3)-H(3B)	109.0	H(14A)-C(14)-H(14C)	109.5
H(3A)-C(3)-H(3B)	107.8	H(14B)-C(14)-H(14C)	109.5
C(13)-C(4)-C(5)	118.74(14)	O(1)-C(15)-C(11)	119.31(15)
C(13)-C(4)-C(3)	121.80(15)	O(1)-C(15)-C(16)	120.26(14)
C(5)-C(4)-C(3)	119.43(15)	C(11)-C(15)-C(16)	119.12(14)
C(6)-C(5)-C(4)	122.13(15)	C(18)-C(16)-C(17)	111.07(15)
C(6)-C(5)-H(5)	118.9	C(18)-C(16)-C(15)	109.30(14)
C(4)-C(5)-H(5)	118.9	C(17)-C(16)-C(15)	113.45(13)
C(5)-C(6)-C(7)	120.29(15)	C(18)-C(16)-C(19)	109.26(14)
C(5)-C(6)-H(6)	119.9	C(17)-C(16)-C(19)	108.78(14)
C(7)-C(6)-H(6)	119.9	C(15)-C(16)-C(19)	104.74(14)
C(6)-C(7)-C(8)	121.65(15)	C(16)-C(17)-H(17A)	109.5
C(6)-C(7)-C(12)	119.11(14)	C(16)-C(17)-H(17B)	109.5
C(8)-C(7)-C(12)	119.23(14)	H(17A)-C(17)-H(17B)	109.5
C(9)-C(8)-C(7)	120.94(15)	C(16)-C(17)-H(17C)	109.5
C(9)-C(8)-H(8)	119.5	H(17A)-C(17)-H(17C)	109.5
C(7)-C(8)-H(8)	119.5	H(17B)-C(17)-H(17C)	109.5
C(8)-C(9)-C(10)	119.92(15)	C(16)-C(18)-H(18A)	109.5
C(8)-C(9)-H(9)	120.0	C(16)-C(18)-H(18B)	109.5
C(10)-C(9)-H(9)	120.0	H(18A)-C(18)-H(18B)	109.5
C(11)-C(10)-C(9)	121.35(16)	C(16)-C(18)-H(18C)	109.5

H(18A)-C(18)-H(18C)	109.5	C(16)-C(19)-H(19C)	109.5
H(18B)-C(18)-H(18C)	109.5	H(19A)-C(19)-H(19C)	109.5
C(16)-C(19)-H(19A)	109.5	H(19B)-C(19)-H(19C)	109.5
C(16)-C(19)-H(19B)	109.5		
H(19A)-C(19)-H(19B)	109.5		

Bond lengths [Å] and angles [°] for Compound **5**.

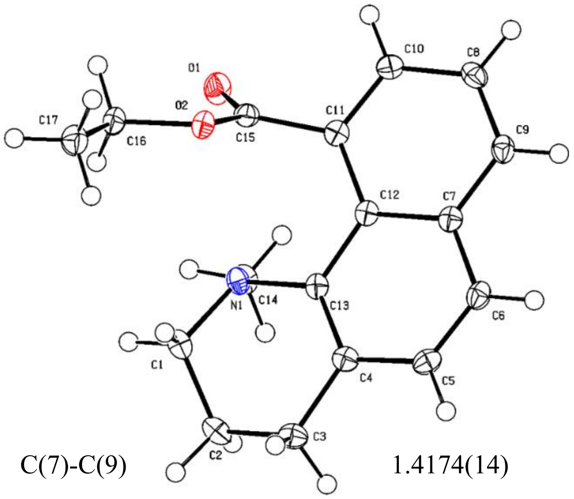

O(1)-C(15)	1.2100(13)	C(7)-C(9)	1.4174(14)
O(2)-C(15)	1.3439(12)	C(7)-C(12)	1.4244(13)
O(2)-C(16)	1.4504(12)	C(8)-C(9)	1.3665(15)
N(1)-C(13)	1.4268(12)	C(8)-C(10)	1.4087(14)
N(1)-C(14)	1.4698(13)	C(8)-H(8)	0.9500
N(1)-C(1)	1.4703(13)	C(9)-H(9)	0.9500
C(1)-C(2)	1.5277(15)	C(10)-C(11)	1.3777(14)
C(1)-H(1A)	0.9900	C(10)-H(10)	0.9500
C(1)-H(1B)	0.9900	C(11)-C(12)	1.4295(14)
C(2)-C(3)	1.5222(14)	C(11)-C(15)	1.4981(14)
C(2)-H(2A)	0.9900	C(12)-C(13)	1.4341(14)
C(2)-H(2B)	0.9900	C(14)-H(14A)	0.9800
C(3)-C(4)	1.5104(14)	C(14)-H(14B)	0.9800
C(3)-H(3A)	0.9900	C(14)-H(14C)	0.9800
C(3)-H(3B)	0.9900	C(16)-C(17)	1.5049(15)
C(4)-C(13)	1.3826(14)	C(16)-H(16A)	0.9900
C(4)-C(5)	1.4167(14)	C(16)-H(16B)	0.9900
C(5)-C(6)	1.3623(15)	C(17)-H(17A)	0.9800
C(5)-H(5)	0.9500	C(17)-H(17B)	0.9800
C(6)-C(7)	1.4188(14)	C(17)-H(17C)	0.9800
C(6)-H(6)	0.9500		

C(15)-O(2)-C(16)	114.24(8)	C(13)-N(1)-C(14)	112.96(8)

C(13)-N(1)-C(1)	112.75(8)	C(8)-C(9)-H(9)	119.5
C(14)-N(1)-C(1)	112.05(8)	C(7)-C(9)-H(9)	119.5
N(1)-C(1)-C(2)	111.48(8)	C(11)-C(10)-C(8)	121.02(9)
N(1)-C(1)-H(1A)	109.3	C(11)-C(10)-H(10)	119.5
C(2)-C(1)-H(1A)	109.3	C(8)-C(10)-H(10)	119.5
N(1)-C(1)-H(1B)	109.3	C(10)-C(11)-C(12)	120.45(9)
C(2)-C(1)-H(1B)	109.3	C(10)-C(11)-C(15)	116.72(9)
H(1A)-C(1)-H(1B)	108.0	C(12)-C(11)-C(15)	122.68(9)
C(3)-C(2)-C(1)	109.43(8)	C(7)-C(12)-C(11)	117.58(9)
C(3)-C(2)-H(2A)	109.8	C(7)-C(12)-C(13)	119.21(9)
C(1)-C(2)-H(2A)	109.8	C(11)-C(12)-C(13)	123.21(9)
C(3)-C(2)-H(2B)	109.8	C(4)-C(13)-N(1)	122.14(9)
C(1)-C(2)-H(2B)	109.8	C(4)-C(13)-C(12)	120.03(9)
H(2A)-C(2)-H(2B)	108.2	N(1)-C(13)-C(12)	117.82(8)
C(4)-C(3)-C(2)	112.44(8)	N(1)-C(14)-H(14A)	109.5
C(4)-C(3)-H(3A)	109.1	N(1)-C(14)-H(14B)	109.5
C(2)-C(3)-H(3A)	109.1	H(14A)-C(14)-H(14B)	109.5
C(4)-C(3)-H(3B)	109.1	N(1)-C(14)-H(14C)	109.5
C(2)-C(3)-H(3B)	109.1	H(14A)-C(14)-H(14C)	109.5
H(3A)-C(3)-H(3B)	107.8	H(14B)-C(14)-H(14C)	109.5
C(13)-C(4)-C(5)	119.16(9)	O(1)-C(15)-O(2)	123.81(9)
C(13)-C(4)-C(3)	122.19(9)	O(1)-C(15)-C(11)	124.02(9)
C(5)-C(4)-C(3)	118.65(9)	O(2)-C(15)-C(11)	111.87(8)
C(6)-C(5)-C(4)	121.84(9)	O(2)-C(16)-C(17)	107.96(8)
C(6)-C(5)-H(5)	119.1	O(2)-C(16)-H(16A)	110.1
C(4)-C(5)-H(5)	119.1	C(17)-C(16)-H(16A)	110.1
C(5)-C(6)-C(7)	120.41(9)	O(2)-C(16)-H(16B)	110.1
C(5)-C(6)-H(6)	119.8	C(17)-C(16)-H(16B)	110.1
C(7)-C(6)-H(6)	119.8	H(16A)-C(16)-H(16B)	108.4
C(9)-C(7)-C(6)	121.56(9)	C(16)-C(17)-H(17A)	109.5
C(9)-C(7)-C(12)	119.77(9)	C(16)-C(17)-H(17B)	109.5
C(6)-C(7)-C(12)	118.66(9)	H(17A)-C(17)-H(17B)	109.5
C(9)-C(8)-C(10)	119.60(9)	C(16)-C(17)-H(17C)	109.5
C(9)-C(8)-H(8)	120.2	H(17A)-C(17)-H(17C)	109.5
C(10)-C(8)-H(8)	120.2	H(17B)-C(17)-H(17C)	109.5
C(8)-C(9)-C(7)	120.99(9)		

Bond lengths [Å] and angles [°] for Compound **6**.

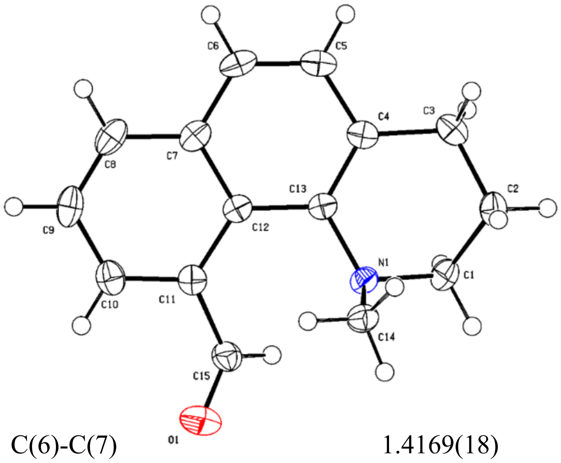

O(1)-C(15)	1.2098(15)	C(6)-C(7)	1.4169(18)
N(1)-C(13)	1.4276(14)	C(7)-C(8)	1.4181(17)
N(1)-C(14)	1.4665(15)	C(7)-C(12)	1.4266(16)
N(1)-C(1)	1.4680(15)	C(8)-C(9)	1.365(2)
C(1)-C(2)	1.5239(17)	C(9)-C(10)	1.4024(18)
C(2)-C(3)	1.5240(17)	C(10)-C(11)	1.3799(17)
C(3)-C(4)	1.5104(17)	C(11)-C(12)	1.4319(16)
C(4)-C(13)	1.3824(16)	C(11)-C(15)	1.4879(16)
C(4)-C(5)	1.4166(17)	C(12)-C(13)	1.4331(16)
C(5)-C(6)	1.3579(19)		

C(13)-N(1)-C(14)	112.84(9)	C(9)-C(8)-C(7)	120.95(11)
C(13)-N(1)-C(1)	112.90(9)	C(8)-C(9)-C(10)	119.69(11)
C(14)-N(1)-C(1)	112.61(9)	C(11)-C(10)-C(9)	121.57(12)
N(1)-C(1)-C(2)	112.35(10)	C(10)-C(11)-C(12)	119.99(11)
C(1)-C(2)-C(3)	108.79(10)	C(10)-C(11)-C(15)	115.78(11)
C(4)-C(3)-C(2)	113.55(10)	C(12)-C(11)-C(15)	123.72(10)
C(13)-C(4)-C(5)	118.85(11)	C(7)-C(12)-C(11)	117.79(10)
C(13)-C(4)-C(3)	122.25(10)	C(7)-C(12)-C(13)	118.52(10)
C(5)-C(4)-C(3)	118.90(10)	C(11)-C(12)-C(13)	123.70(10)
C(6)-C(5)-C(4)	121.80(11)	C(4)-C(13)-N(1)	121.49(10)
C(5)-C(6)-C(7)	120.58(11)	C(4)-C(13)-C(12)	120.69(10)
C(6)-C(7)-C(8)	121.23(11)	N(1)-C(13)-C(12)	117.82(10)
C(6)-C(7)-C(12)	118.95(11)	O(1)-C(15)-C(11)	123.22(12)
C(8)-C(7)-C(12)	119.80(11)		

Crystal data and structure refinement for Compound **4**.

CCDC deposit number	2355953	
Empirical formula	$C_{19}H_{23}N O$	
Formula weight	281.38	
Temperature	100(2) K	
Wavelength	0.71073 Å	
Crystal system	Triclinic	
Space group	P-1	
Unit cell dimensions	a = 8.0501(5) Å	α= 65.739(2)°.
	b = 14.6258(8) Å	β= 80.565(2)°.
	c = 15.2351(9) Å	γ = 74.190(2)°.
Volume	1570.72(16) $Å^3$	
Z	4	
Density (calculated)	1.190 Mg/m^3	
Absorption coefficient	0.073 mm^{-1}	
F(000)	608	
Crystal size	0.415 x 0.294 x 0.106 mm^3	
Theta range for data collection	2.529 to 26.050°.	
Index ranges	-9<=h<=9, -18<=k<=18, -18<=l<=18	
Reflections collected	60829	
Independent reflections	6156 [R(int) = 0.0676]	
Completeness to theta = 25.242°	99.9 %	
Refinement method	Full-matrix least-squares on F^2	
Data / restraints / parameters	6156 / 0 / 387	
Goodness-of-fit on F^2	1.023	
Final R indices [I>2sigma(I)]	R1 = 0.0470, wR2 = 0.1144	
R indices (all data)	R1 = 0.0621, wR2 = 0.1230	
Extinction coefficient	n/a	
Largest diff. peak and hole	0.375 and -0.210 e.$Å^{-3}$	

Crystal data and structure refinement for Compound **5**.

CCDC deposit number	2355952	
Empirical formula	$C_{17}H_{19}NO_2$	
Formula weight	269.33	
Temperature	100(2) K	
Wavelength	0.71073 Å	
Crystal system	Monoclinic	
Space group	$P2_1/c$	
Unit cell dimensions	a = 12.6318(6) Å	α= 90°.
	b = 6.7563(3) Å	β= 93.991(2)°.
	c = 16.2925(8) Å	γ = 90°.
Volume	1387.10(11) $Å^3$	
Z	4	
Density (calculated)	1.290 Mg/m^3	
Absorption coefficient	0.084 mm^{-1}	
F(000)	576	
Crystal size	0.570 x 0.293 x 0.222 mm^3	
Theta range for data collection	2.506 to 26.060°.	
Index ranges	-15<=h<=15, -8<=k<=8, -20<=l<=20	
Reflections collected	40393	
Independent reflections	2741 [R(int) = 0.0438]	
Completeness to theta = 25.242°	100.0 %	
Refinement method	Full-matrix least-squares on F^2	
Data / restraints / parameters	2741 / 0 / 184	
Goodness-of-fit on F^2	1.027	
Final R indices [I>2sigma(I)]	R1 = 0.0326, wR2 = 0.0901	
R indices (all data)	R1 = 0.0349, wR2 = 0.0924	
Extinction coefficient	0.0150(18)	
Largest diff. peak and hole	0.230 and -0.173 e.$Å^{-3}$	

Crystal data and structure refinement for Compound **6**.

CCDC deposit number	2355951	
Empirical formula	$C_{15}H_{15}NO$	
Formula weight	225.28	
Temperature	100(2) K	
Wavelength	0.71073 Å	
Crystal system	Monoclinic	
Space group	$P2_1/n$	
Unit cell dimensions	a = 8.4652(2) Å	α= 90°.
	b = 9.6199(3) Å	β= 105.1990(10)°.
	c = 14.5468(4) Å	γ = 90°.
Volume	1143.17(5) $Å^3$	
Z	4	
Density (calculated)	1.309 Mg/m^3	
Absorption coefficient	0.082 mm^{-1}	
F(000)	480	
Crystal size	0.220 x 0.110 x 0.040 mm^3	
Theta range for data collection	2.535 to 26.022°.	
Index ranges	-10<=h<=10, -11<=k<=11, -17<=l<=17	
Reflections collected	29187	
Independent reflections	2240 [R(int) = 0.0362]	
Completeness to theta = 25.242°	99.8 %	
Refinement method	Full-matrix least-squares on F^2	
Data / restraints / parameters	2240 / 0 / 155	
Goodness-of-fit on F^2	1.095	
Final R indices [I>2sigma(I)]	R1 = 0.0372, wR2 = 0.0996	
R indices (all data)	R1 = 0.0414, wR2 = 0.1045	
Extinction coefficient	n/a	
Largest diff. peak and hole	0.232 and -0.176 e.$Å^{-3}$	

Table S1. Calculated excitation ($S_0 \rightarrow S_n$) data for **4–6**.

	Excited state	Configuration	E (eV)	Abs. (nm)	f_{osc}
**4**	S_1	H→L (69%), H-1→L+1 (10%)	4.13	300	0.159
	S_2	H-3→L+3 (17%), H-3→L+4 (-11%), H-2→L (-11%), H-2→L+1 (12%), H-1→L (-27%), H→L+1 (57%)	4.26	291	0.025
	S_4	H-2→L+1 (-13%), H-1→L (41%), H→L+1 (25%), H→L+3 (-36%), H→L+4 (21%)	4.97	250	0.071
**5**	S_1	H→L (69%)	4.05	306	0.152
	S_2	H-2→L (-16%), H-1→L (32%) H→L+1 (59%)	4.29	289	0.026
	S_3	H-2→L (24%), H-1→L (52%), H→L+1 (-22%), H→L+5 (-14%) H→L+6 (-21%)	5.05	246	0.188
**6**	S_1	H→L (68%), H→L+1 (-11%)	3.77	329	0.116
	S_2	H-3→L (44%), H-3→L+1 (-13%), H-3→L+2 (28%), H-2→L (-32%), H-2→L+2 (-16%)	4.11	302	0.011
	S_3	H-1→L (35%), H→L (11%), H→L+1 (58%)	4.68	265	0.074

Calculated from the optimized ground state structures using the TD-SCF DFT CAM-B3LYP method with the 6-311G + (2d,p) basis set and the IEFPCM solvent model for toluene.

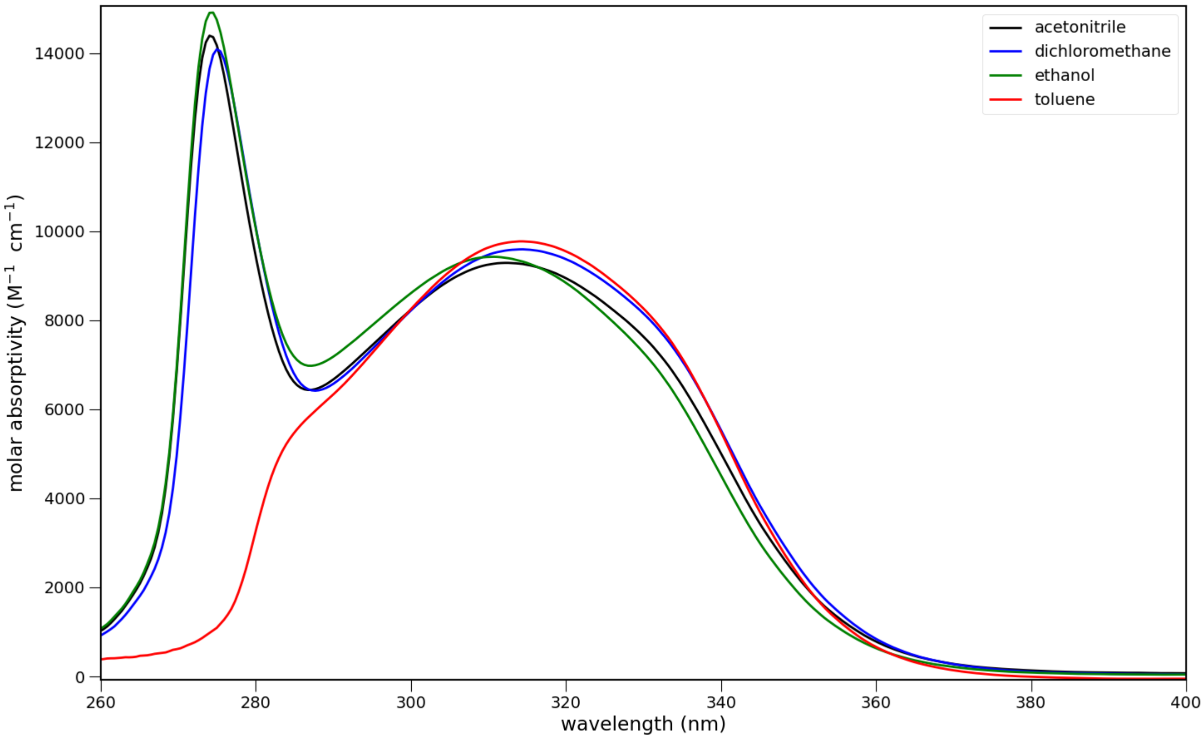

Figure S1. Absorption spectra of **4** in acetonitrile, dichloromethane, ethanol, and toluene.

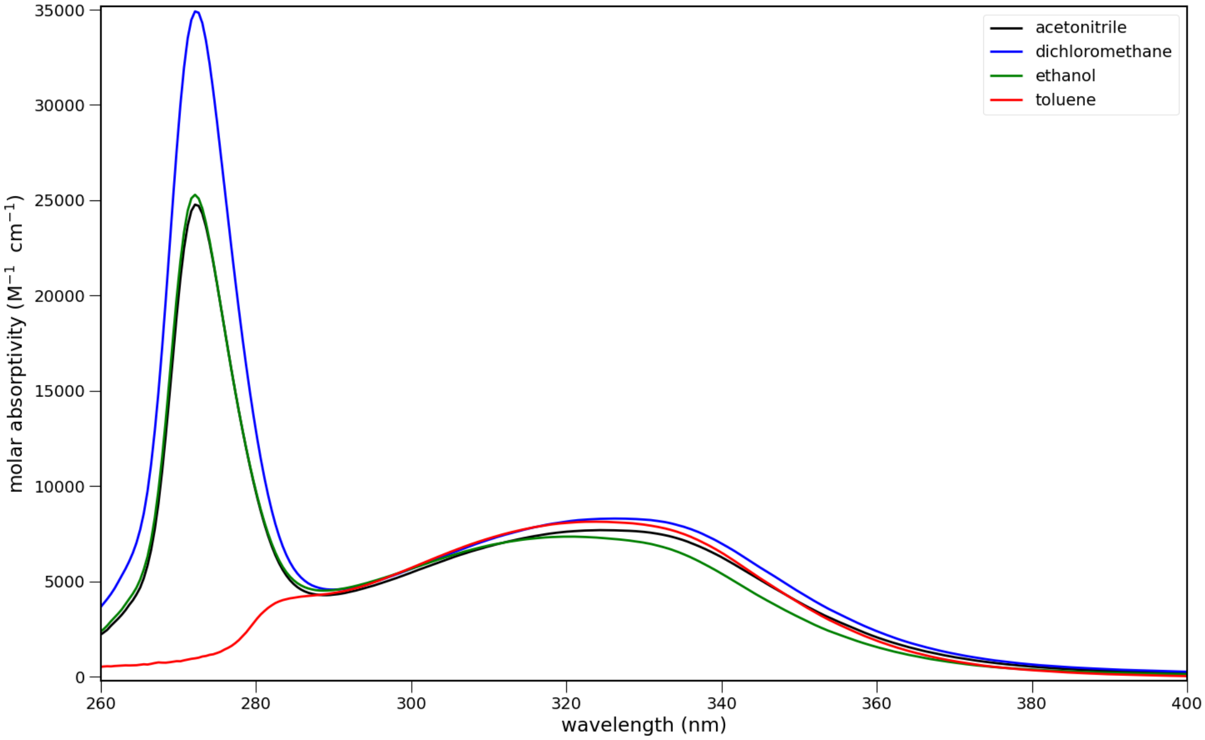

Figure S2. Absorption spectra of **5** in acetonitrile, dichloromethane, ethanol, and toluene.

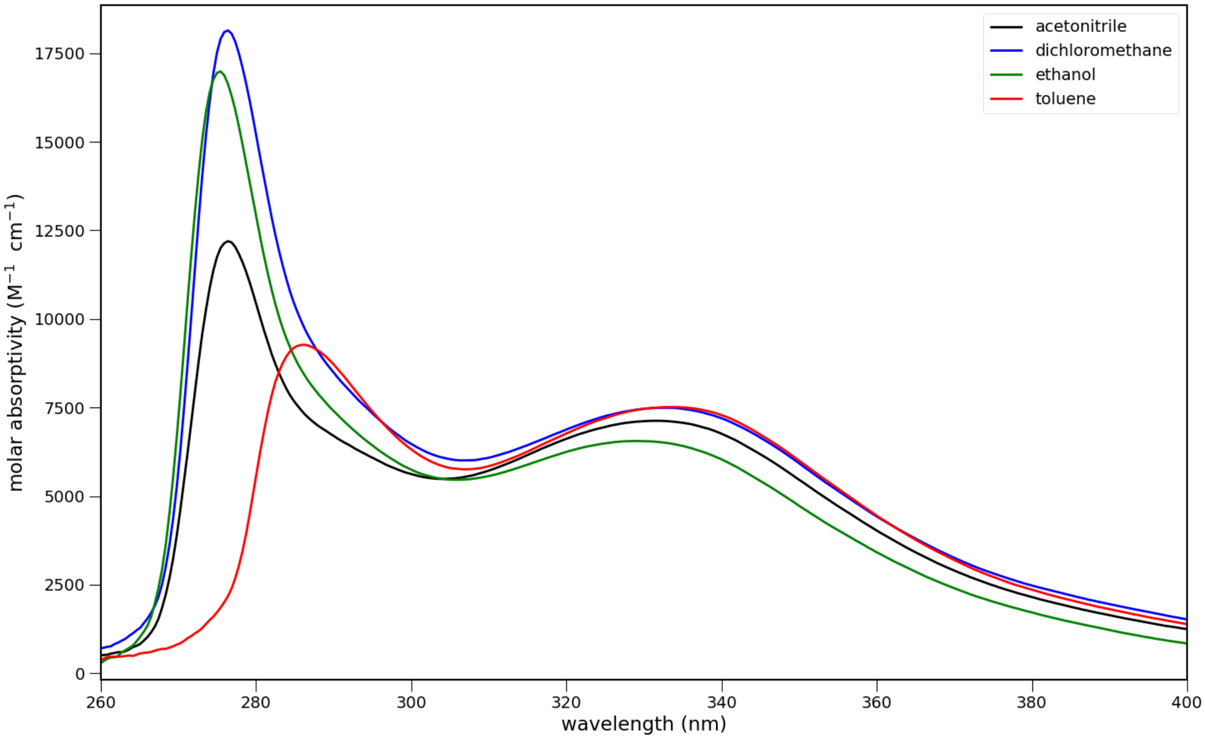

Figure S3. Absorption spectra of **6** in acetonitrile, dichloromethane, ethanol, and toluene.

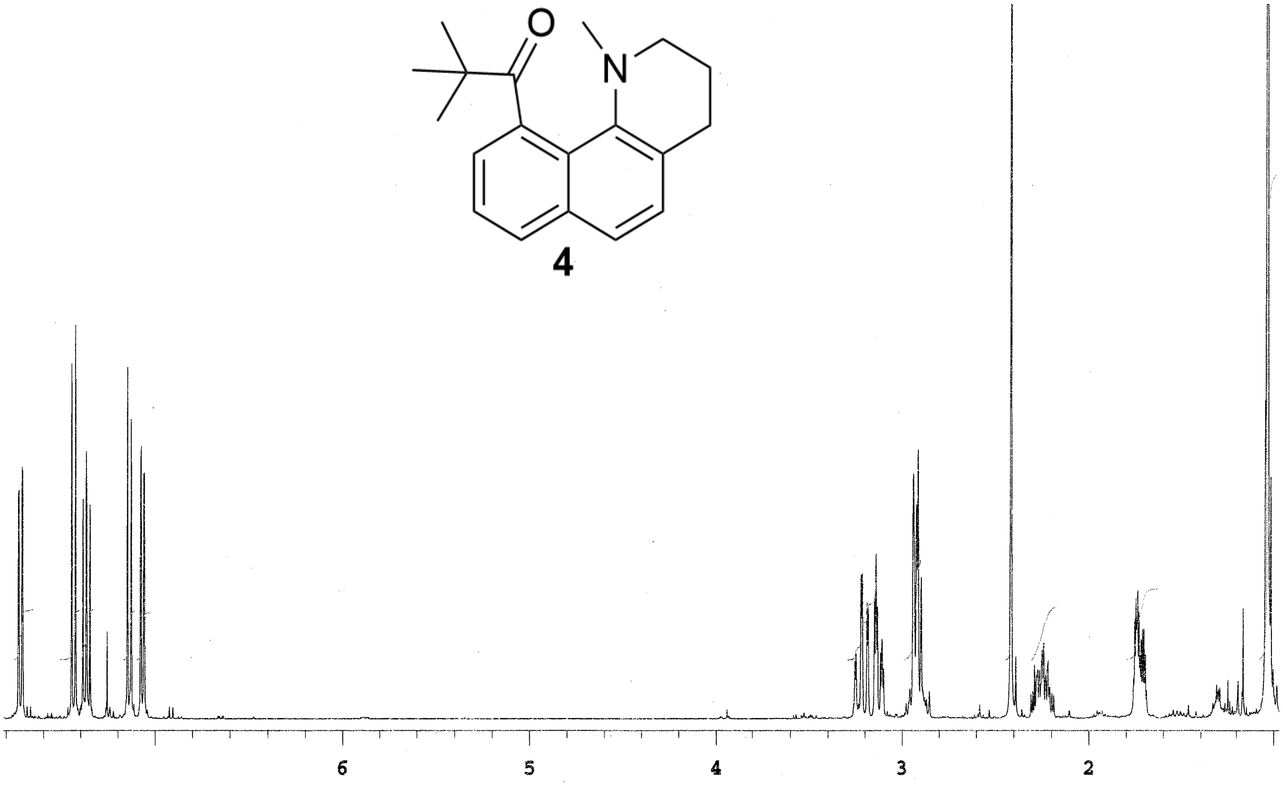

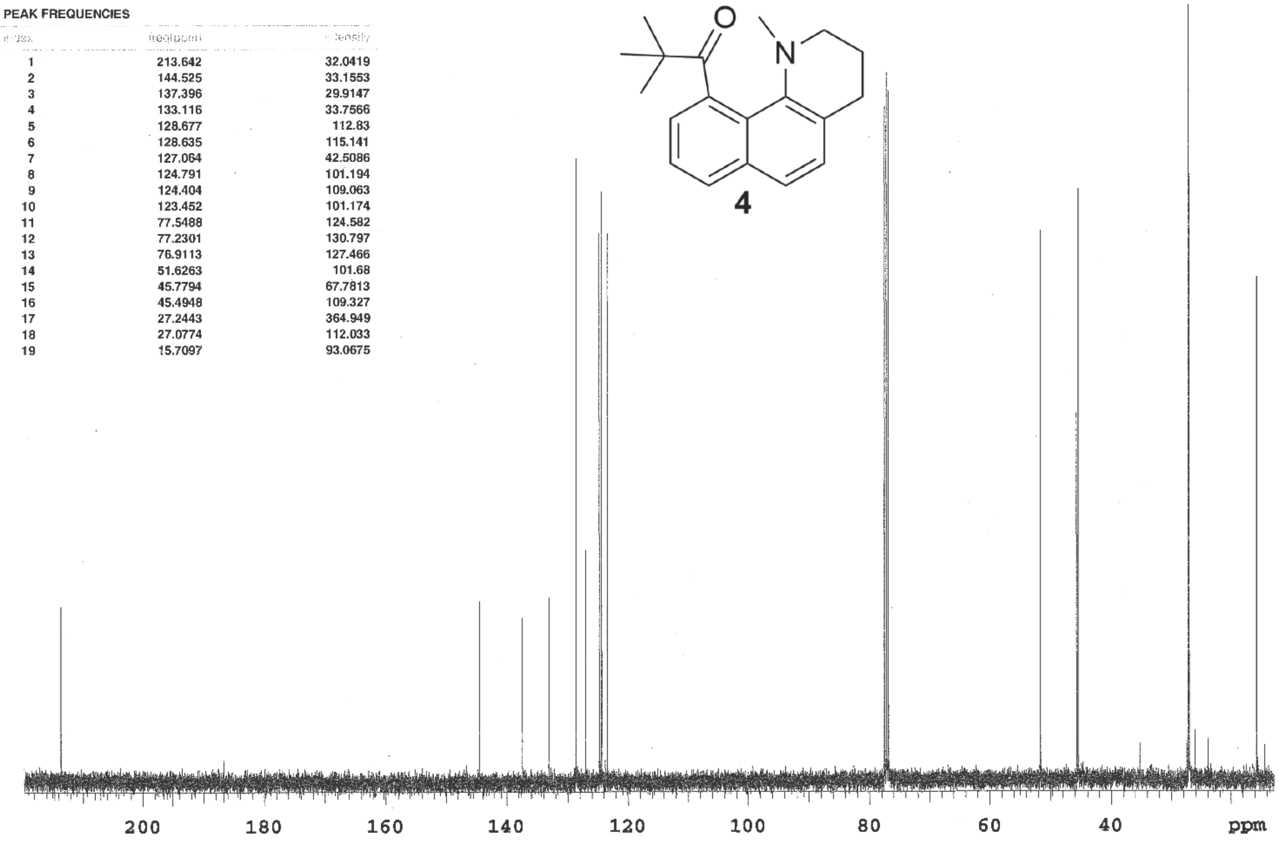

PEAK FREQUENCIES

index	freq(ppm)	intensity
1	213.642	32.0419
2	144.525	33.1553
3	137.396	29.9147
4	133.116	33.7566
5	128.677	112.83
6	128.635	115.141
7	127.064	42.5086
8	124.791	101.194
9	124.404	109.063
10	123.452	101.174
11	77.5488	124.582
12	77.2301	130.797
13	76.9113	127.466
14	51.6263	101.68
15	45.7794	67.7813
16	45.4948	109.327
17	27.2443	364.949
18	27.0774	112.033
19	15.7097	93.0675

Figure S4. ^{1}H (top) and ^{13}CNMR spectra of **4** in $CDCl_3$.

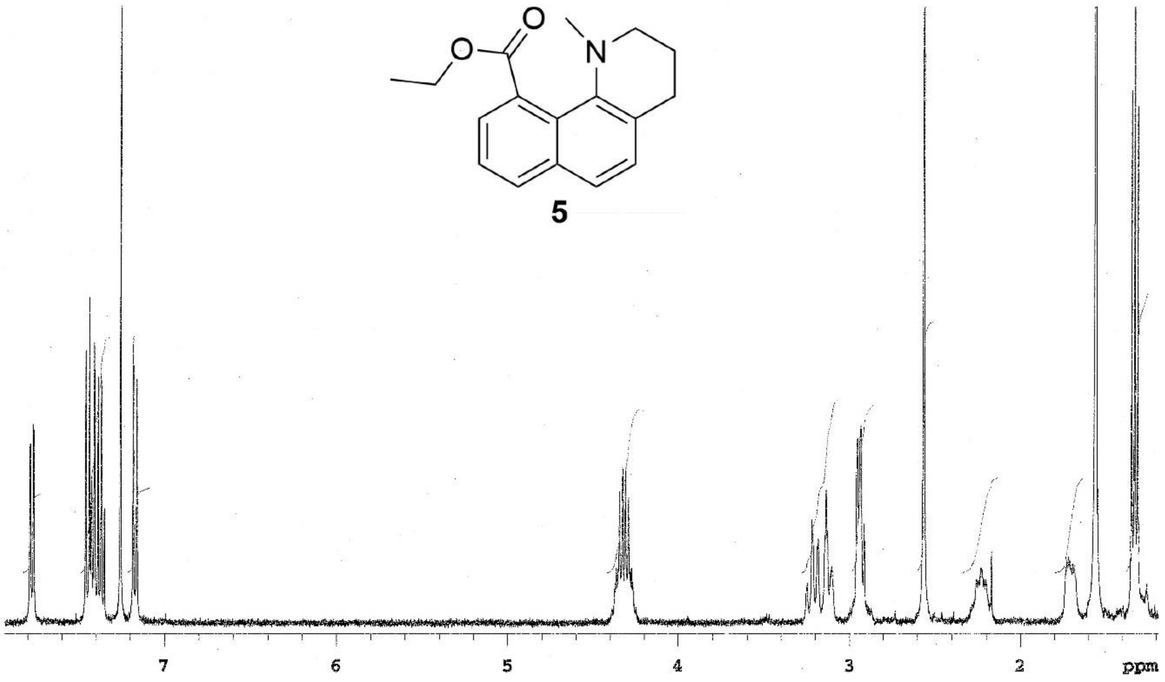

PEAK FREQUENCIES

index	freq(ppm)	intensity
1	172.059	19.2925
2	144.482	22.1962
3	133.608	41.436
4	130.792	32.824
5	129.54	108.5
6	128.683	91.0175
7	127.954	37.0049
8	126.846	22.9241
9	125.366	92.2133
10	124.319	99.0338
11	122.839	88.9484
12	77.4524	33.9632
13	77.1337	34.2667
14	76.8149	32.7536
15	61.0004	74.3997
16	50.3689	73.9294
17	45.4287	99.2046
18	27.0038	74.0066
19	15.5223	72.0749
20	14.3764	89.5879

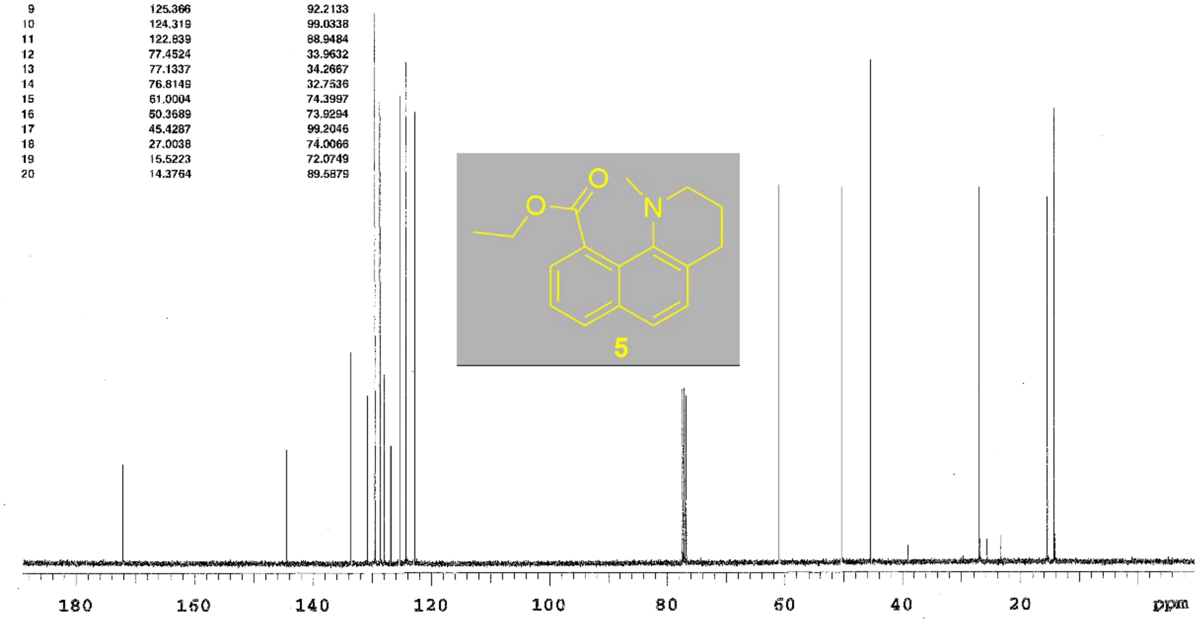

Figure S5. ^{1}H (top) and ^{13}CNMR spectra of **5** in $CDCl_3$.

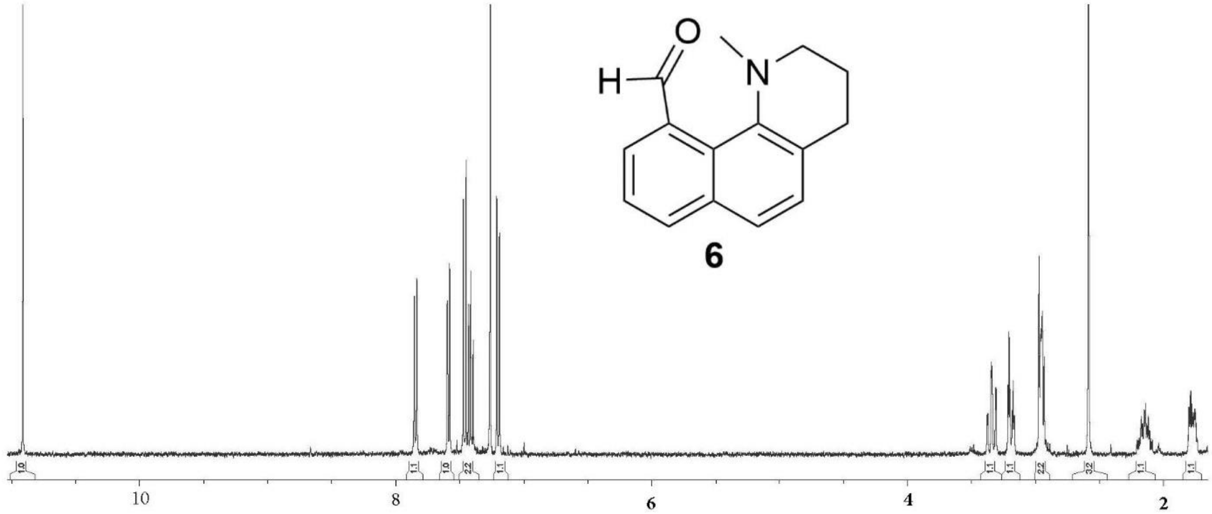

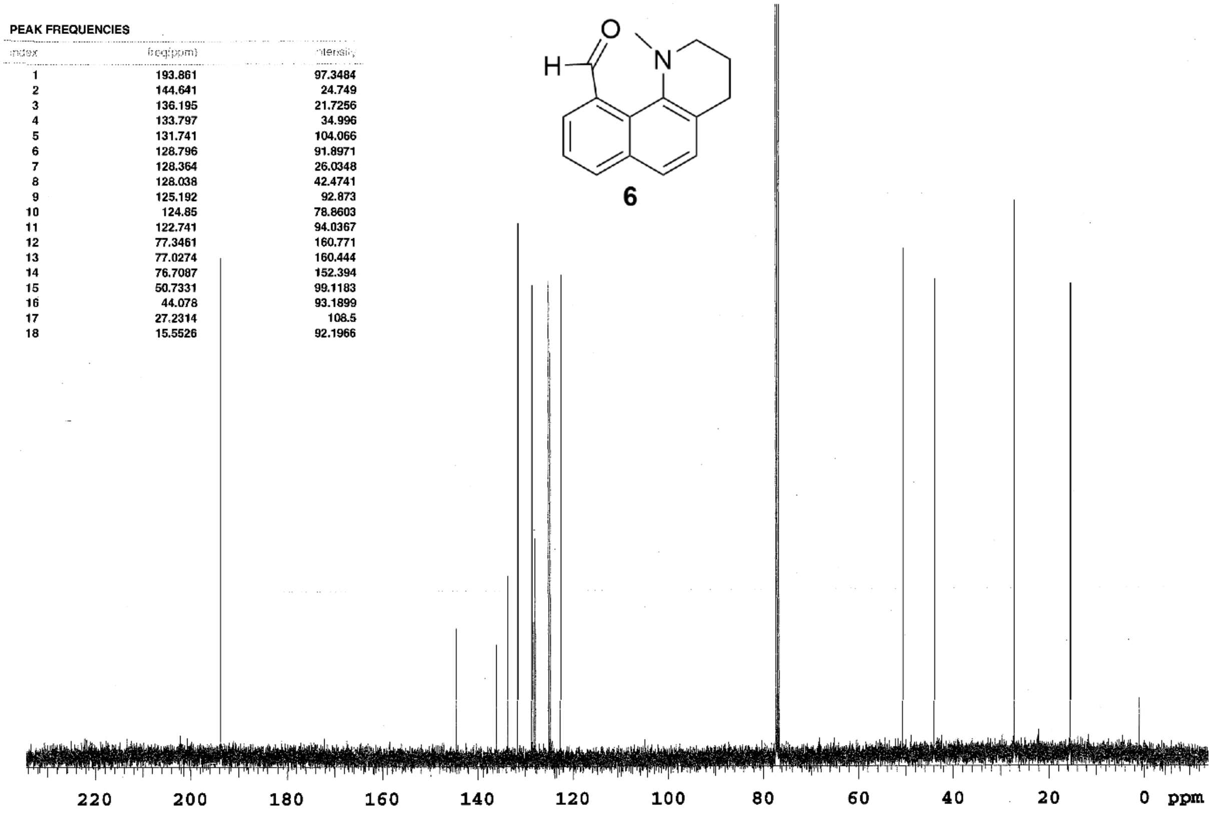

PEAK FREQUENCIES

index	freq(ppm)	intensity
1	193.861	97.3484
2	144.641	24.749
3	136.195	21.7256
4	133.797	34.996
5	131.741	104.066
6	128.796	91.8971
7	128.364	26.0348
8	128.038	42.4741
9	125.192	92.873
10	124.85	78.8603
11	122.741	94.0367
12	77.3461	160.771
13	77.0274	160.444
14	76.7087	152.394
15	50.7331	99.1183
16	44.078	93.1899
17	27.2314	108.5
18	15.5526	92.1966

Figure S6. ^{1}H (top) and ^{13}CNMR spectra of **6** in $CDCl_3$.
